# MOTS-c Functionally Prevents Metabolic Disorders

**DOI:** 10.3390/metabo13010125

**Published:** 2023-01-13

**Authors:** Yue Gao, Xinran Wei, Pingying Wei, Huijie Lu, Luying Zhong, Jie Tan, Hongbo Liu, Zheng Liu

**Affiliations:** 1 College of Medical Laboratory Science, Guilin Medical University, Guilin 541004, China; 2 Department of Laboratory Medicine, The Second Affiliated Hospital of Guilin Medical University, Guilin 541199, China; 3 Guangxi Key Laboratory of Brain and Cognitive Neuroscience, Guilin Medical University, Guilin 541199, China; 4 Guangxi Health Commission Key Laboratory of Glucose and Lipid Metabolism Disorders, Guilin 541199, China

**Keywords:** MOTS-c, insulin resistance, obesity, muscle function, bone metabolism, immune regulation, aging

## Abstract

Mitochondrial-derived peptides are a family of peptides encoded by short open reading frames in the mitochondrial genome, which have regulatory effects on mitochondrial functions, gene expression, and metabolic homeostasis of the body. As a new member of the mitochondrial-derived peptide family, mitochondrial open reading frame of the 12S rRNA-c (MOTS-c) is regarding a peptide hormone that could reduce insulin resistance, prevent obesity, improve muscle function, promote bone metabolism, enhance immune regulation, and postpone aging. MOTS-c plays these physiological functions mainly through activating the AICAR-AMPK signaling pathways by disrupting the folate-methionine cycle in cells. Recent studies have shown that the above hormonal effect can be achieved through MOTS-c regulating the expression of genes such as GLUT4, STAT3, and IL-10. However, there is a lack of articles summarizing the genes and pathways involved in the physiological activity of MOTS-c. This article aims to summarize and interpret the interesting and updated findings of MOTS-c-associated genes and pathways involved in pathological metabolic processes. Finally, it is expected to develop novel diagnostic markers and treatment approaches with MOTS-c to prevent and treat metabolic disorders in the future.

## 1. Introduction 

The mitochondrion is a double membrane-bound half-independent organelle in the cell. It is the most important cellular sources of reactive oxygen species (ROS) and the main production site of ATP. As an energy factory of cellular activities, mitochondria not only provide energy but also respond to metabolic stress and regulates the metabolic balance in the cells [1]. The human mitochondrial genome (mtDNA) is a small 16,569 bp DNA circle that has 37 genes, encoding for 22 tRNA, 2 rRNA, 13 mitochondrial respiratory chain and oxidative phosphorylation-related protein subunits [2]. Mitochondrial-derived peptides (MDPs) are small peptides encoded by the short open reading frame (ORF) of mitochondrial DNA. To date, three types of MDPs, namely humanin, small humanin-like peptides (SHLPs), and mitochondrial open reading frame of the 12S rRNA-c (MOTS-c), have been identified [3,4,5]. However, the physiological function of MDPs is largely unknown. 

**Figure 1:**
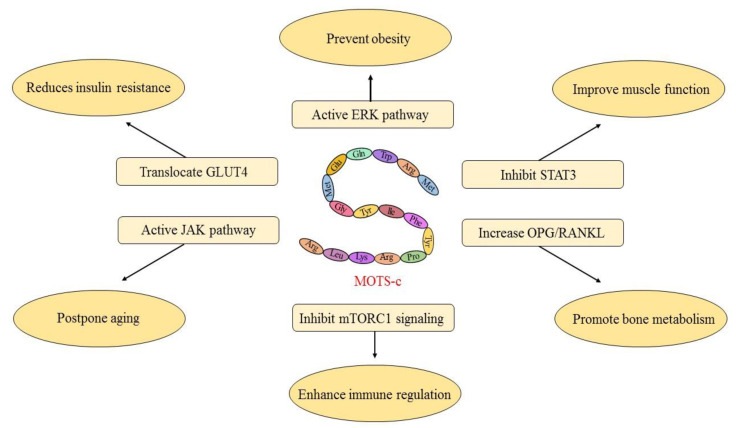
The main physiological function of MOTS-C is to reduce insulin resistance, prevent obesity, improve muscle function, promote bone metabolism, enhance immune regulation, and postpone aging. These physiological functions are primarily accomplished by translocating GLUT4, activing the ERK pathway, inhibiting STAT3, increasing OPG/RANKL, inhibiting mTORC1 signaling, and activing the JAK pathway, respectively.

MOTS-c is detected in various tissues and organs [5]. Previous studies indicate that MOTS-c acts in a systemic manner, which is hence qualified as a peptide hormone [6]. According to some studies, MOTS-c acts as a retrograde signaling molecule of mitochondria and transmits messages to the nucleus [7]. Therefore, we believe that MOTS-c primarily regulates gene expression by entering the nucleus, where it then affects the physiological metabolism of the body to play its role. We summarize the reported effects of MOTS-c on body metabolism into six aspects, including insulin resistance reduction, obesity prevention, muscle function improvement, bone metabolism promotion, immune regulation enhancement, and aging postponement in this article (Figure 1). The altered expression of genes and pathways involved in the above effect is also reviewed in the hope of providing support for further investigation of MOTS-c. 

## 2. An Overview of MOTS-c

### 2.1. Discovery of MOTS-c 

MOTS-c is a new member of the mitochondrial polypeptide family, encoded by the 12S rRNA short ORF of mtDNA. MOTS-c was first discovered by Lee et al. in 2015 [5]. They looked for potential ORF in human 12S rRNA and found one that could be translated into a 16-amino-acid peptide, which they termed MOTS-c. The primary structure of MOTS-c is Met-Arg-Trp-Gln-Glu-Met-Gly-Tyr-Ile-Phe-Tyr-Pro-Arg-Lys-Leu-Arg, and the first 11 amino acid residues are highly conserved. Lee et al. also found that MOTS-c was widely expressed in numerous tissues, including the brain, heart, liver, muscles, testes, kidney, spleen, large intestine and small intestine. MOTS-c treatment in mice prevented age-dependent and high-fat-diet-induced insulin resistance, as well as diet-induced obesity [5]. 

MOTS-c is an adaptive signaling protein that can translocate to the nucleus under metabolic stress [7]. MOTS-c engages in intracellular nuclear-mitochondrial signal transmission, regulates nuclear gene expression, and maintains mitochondrial function and cell resilience [7]. MOTS-c, on the other hand, has a hormonal effect. By controlling the synthesis of antioxidant response kinases within a range and activating related metabolic pathways, it affects glucose, lipid and bone metabolism [8,9]. Since MOTS-c plays an important role in the pathological metabolic processes of several metabolic disorders, MOTS-c is expected to become a diagnostic marker and an effective treatment approach for these disorders [10]. 

### 2.2. Molecular Mechanism of MOTS-c Regulating Cell Metabolism

**Figure 2:**
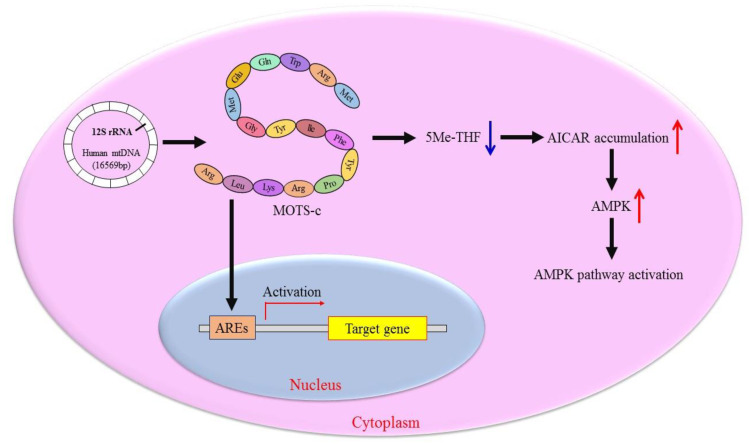
The main physiological functions of MOTS-c in cells. MOTS-c is encoded by the 12S rRNA short open reading frame of mtDNA. It disrupts the folate-methionine cycle and consumes 5Me-THF under metabolic stress, leading to higher amounts of endogenous AICAR. An accumulated AICAR activates the AMPK pathway. Alternatively, MOTS-c binds to the antioxidant responsive elements (AREs) in nucleus, which regulate the ARE-related gene expression.

Mitochondria communicate with the cell nucleus via a complex communication system. The dynamic dialogue between the two compartments is critical for maintaining cellular homeostasis in case of metabolic stress [11]. In fact, the expression of mitochondrial gene not only passively accepts the regulation of nuclear DNA coding elements but also actively influences the expression of nuclear genes. When the cells are under metabolic stress, MOTS-c, an active mitochondrial-encoded signal factor, can be rapidly transferred from mitochondria to the nucleus and regulates nuclear gene expression in an AMP-activated protein kinase (AMPK)-dependent manner [7]. MOTS-c can also bind to the antioxidant-responsive elements (AREs) in the nucleus, which regulate the interaction of transcription factors, thereby controlling the nuclear gene expression (Figure 2) [7]. This stress-responsive nuclear translocation of MOTS-c helps the body stimulate the corresponding metabolism and maintain cell metabolic balance. 

MOTS-c regulates cell metabolism primarily through the AICAR (5-aminoimidazole-4-carboxamide ribonucleotide)-AMPK pathway, which controls glycolysis, mitochondrial activity, and fatty acid oxidation [12]. As a signal hub, the function of AMPK is to balance fuel consumption and energy requirements [13]. MOTS-c has been demonstrated to disrupt the folate-methionine cycle and impede the new purine de novo synthesis, which consumes 5-methyltetrahydro-folate (5Me-THF), resulting in higher amounts of endogenous AICAR [5,12]. An accumulated AICAR activates AMPK to accelerate fatty acid oxidation via the phosphorylation of acetyl-CoA carboxylase (ACC) (Figure 2) [14]. The effect of MOTS-c on cells includes enhancing insulin sensitivity, improving glucose tolerance, and increasing glucose uptake [7,15]. MOTS-c can increase the level of the metabolite nicotinamide adenine dinucleotide (NAD). MOTS-c also can activate glycolysis and the pentose phosphate pathway, which regulates intracellular metabolic control in response to metabolic stress [5]. 

## 3. MOTS-c Inhibits Pathological Metabolic Processes

### 3.1. MOTS-c Reduces Insulin Resistance

Insulin resistance is defined as impaired signal transduction and biological actions in response to insulin stimulation, resulting in a decrease in the ability of insulin to increase glucose uptake and utilization [16]. Insulin resistance is a risk factor for non-alcoholic steatohepatitis, metabolic syndrome, and type 2 diabetes (T2D) [17,18,19]. MOTS-c has been demonstrated to increase insulin sensitivity in mice by preventing fat accumulation through reducing the pathways of sphingolipid metabolism, monoacylglycerol metabolism, and dicarboxylate metabolism [20]. MOTS-c treatment reversed age-dependent insulin resistance in mouse skeletal muscle, demonstrating that the occurrence of insulin resistance is related to MOTS-c [5,21]. MOTS-c can correct the diabetes-induced abnormal cardiac structures and functions. MOTS-c mimics exercise-induced cardio-protection in diabetic rats by activating NRG1-ErbB4 signaling pathway [22].

By increasing the expression of mitochondrial biogenesis genes TFAM, COX4, and NRF1, which are markers for mitochondrial biogenesis, MOTS-c induces GLUT4 translocation and promotes glucose uptake [23]. MOTS-c also improve the glucose metabolism of insulin-resistant rats fed with high-fat diets. The mechanism is that MOTS-c increases the AMPK activity and enhances the insulin sensitivity of skeletal muscle cells, resulting in improved glucose metabolism [5]. This result indicates that MOTS-c can be employed as a new insulin sensitizer, which is helpful in treating T2D. Interestingly, a C allele of the East Asian-specific m.1382A > C polymorphism (rs111033358) leads to the MOTS-c K14Q, which contributes to the risk of T2D in sedentary males [24]. It suggests that the function of MOTS-c has an exercise-like effect on T2D. Table 1 shows the substitution of lysine to glutamine caused by the m.1382A > C polymorphism.

### 3.2. MOTS-c Prevents Obesity

Obesity is a risk factor for insulin resistance and hyperinsulinemia [25]. MOTS-c in the maternal blood serum of obese women significantly increases compared to healthy women [6]. It has been found in previous studies that MOTS-c injections in the mouse could reduce the level of IL-6 and TNF-α, two obesity-related inflammatory factors [26,27]. Similarly, the AMPK pathway is activated after MOTS-c is given to high-fat diet-induced mice. The expression of the downstream GLUT4 rises, which reduce the incidence of obesity [5,23]. Additionally, mice treated with MOTS-c significantly decrease plasma lipid and hepatic triacylglycerol levels [5,8]. It is well known that obesity is an independent risk factor for cardiovascular disease, such as vascular calcification. Our research group demonstrated that MOTS-c treatment significantly attenuated vascular calcification by activating the AMPK signaling pathway and suppressing the expression of the AT-1 as well as ET-B receptors [28]. 

It is found in a case–control study that the MOTS-c level of obese male children and adolescents is significantly lower than that of control, and the level of MOTS-c is inversely linked with the level of obesity-related markers [29]. However, this relationship is not observed in the female group. We hypothesize that hormones secreted by ovaries interfere with the expression of MOTS-c. As ovarian functions decline after menopause, less estrogen is secreted, which raises the probability of lipid metabolic disorders and fat deposition. This hypothesis is verified in the following report. When MOTS-c was administered to an ovariectomized mouse model, the expression of genes related to lipid oxidation elevated while that of genes related to lipogenesis was repressed, improving the lipid catabolism of ovariectomized mice [8]. In addition, MOTS-c administration increases the white fat browning to reduce obesity. The process of white fat browning is activated by increasing PGC1α, UCP1, and Dio2 via phosphorylation of the ERK signaling pathway [30].

### 3.3. MOTS-c Improve Muscle Function

Previous research has shown that MOTS-c was naturally muscle-targeting, which could speed up skeletal muscle metabolism while generating energy [31]. MOTS-c increases the level of glycolysis and ATP in dystrophic muscle cells, enhancing the absorption and activity of phosphorodiamidate morpholino oligomer [31]. Additionally, it was found that by giving a long-term combined administration of MOTS-c and phosphorodiamidate morpholino oligomer to Duchenne muscular dystrophy (DMD)-affected mice, their muscular functions significantly improved [31]. Therefore, MOTS-c can be utilized as an adjuvant to enhance the therapeutic effect of oligonucleotide-mediated exon-skipping therapy for insulin-resistance-induced skeletal muscle atrophy [31,32]. Interestingly, MOTS-c interact with STAT3 via the YIFY region to reduce STAT3 transcriptional activity, which enhances myotube formation, suggesting that MOTS-c have a diagnostic and therapeutic promise for muscle-wasting and other energy-deficient muscle illnesses [33]. STAT3 is the first reported gene that directly interacts with MOTS-c. 

Myostatin is a negative regulator of skeletal muscle mass and mediates insulin-resistance-induced skeletal muscle wasting [34,35]. In human circulation, the level of myostatin is adversely linked with plasma MOTS-c [32,36]. Through MOTS-c treatment, the myostatin level decreases in plasma and skeletal muscles. MOTS-c prevents muscle atrophy in diet-induced obese mice by inhibiting the activity of an upstream transcription factor for myostatin and muscle wasting gene, FOXO1 [32]. Serum myostatin has a close relationship with endothelial function. It has been reported that circulating MOTS-c level in patients with coronary endothelial dysfunction declines significantly [37]. Through the APPL1-SIRT1-PGC1α pathway, the production of skeletal muscle MOTS-c is increased by mediating adiponectin signaling [38]. MOTS-c is found to be related to the slow-twitched fiber gene expression [39]. Interestingly, it was found that m.1382A > C polymorphism contributes to the sprint/power performance through regulating skeletal muscle fiber composition [39]. 

### 3.4. MOTS-c Promotes Bone Metabolism

Osteoblasts and osteoclasts are the two most important cells in bone metabolism. Osteoblasts are cells found on bone surfaces that are responsible for bone formation by synthesizing the organic components of the bone matrix and coordinating the mineralization of the skeleton [40]. Osteoclasts are multinucleated cells derived from hematopoietic stem cells or monocyte/macrophage precursor cells that absorb the organic and inorganic compounds released from the impaired bone, during which the degraded compound matrix goes into the bloodstream in the form of Ca^2+^ and so on for recycling [41]. Severe bone formation defects and increased bone resorption can easily lead to metabolic bone diseases such as osteoporosis, rickets, and bone fragility [42,43,44]. Studies have demonstrated that MOTS-c improved bone metabolism abnormalities and acted as an anti-osteoporosis agent by suppressing osteoclast differentiation [9,45]. MOTS-c activate the TGF-β/Smad signaling pathway by up-regulating the expression level of TGF-β1/β2 and Smad7, promoting the synthesis of type I collagen in osteoblasts, and thereby improving osteoporosis [45]. MOTS-c also encourage bone marrow mesenchymal stem cells to develop into osteoblasts and advance the process of bone formation via activating the TGF-β/Smad signaling pathway [45]. 

Several different mechanisms are involved in the regulation of the osteolysis process through MOTS-c. By activating the AMPK pathway and preventing bone marrow-derived macrophages from differentiating into osteoclasts, MOTS-c prevents bone loss caused by estrogen deficiency [9]. MOTS-c inhibited RANKL and up-regulates the rate of OPG/RANKL in osteocytes, leading to inhibition of osteoclastogenesis [9,46]. In addition to the aforementioned pathways, by suppressing inflammation via restraining NF-κB depending on the AMPK-PGC1α-ROS axis pathway and STAT1 pathway, MOTS-c prevents osteoclast development in animal models of osteolysis [46]. These results provide the evidence that MOTS-c has the physiological function of promoting bone metabolism and preventing osteoporosis.

### 3.5. MOTS-c Enhances Immune Regulation

Type 1 diabetes (T1D) is an autoimmune disease characterized by the destruction of pancreatic β-cells, resulting in insufficient insulin secretion [47]. MOTS-c prevents autoimmune β cell destruction by targeting T cells in non-obese diabetic mice [48]. Treatment with MOTS-c for non-obese diabetic mice shows that MOTS-c regulates the T cell phenotype and function through inhibiting TCR/mTORC1 signaling [48]. MOTS-c ameliorated the development of hyperglycemia and reduced islet-infiltrating immune cells [48]. Meanwhile, MOTS-c suppresses autoimmune diabetes, suggesting that MOTS-c could delay the onset of T1D and has the effect of early prevention. MOTS-c was found to be significantly reduced in patients with multiple sclerosis, an autoimmune disease of the central nervous system [49]. These studies indicate that MOTS-c has the physiological function of enhancing immune regulation. 

MOTS-c may also be a potential molecule for the treatment of inflammation-related disorders. MOTS-c protect rat cardiomyocytes from H_2_O_2_-induced inflammation and oxidative stress by suppressing NF-κB and activating the Nrf2-ARE pathway [50]. In a mouse formalin test, MOTS-c could activate the AMPK pathway, which suppressed pro-inflammatory cytokine (TNF-α, IL-1β, and IL-6) production and promoted anti-inflammatory cytokine (IL-10) secretion [26]. In addition, MOTS-c suppressed formalin-induced ERK, JNK, and P38 [26]. Interestingly, MOTS-c improve the survival status of mice during methicillin-resistant staphylococcus aureus infection and promote the AhR/Stat3 signaling pathway by declining pro-inflammatory cytokines (TNF-α, IL-6, and IL-1β) and increasing anti-inflammatory cytokines IL-10 [51]. We hypothesize that the mechanism is that MOTS-c induces the production of antimicrobial factors and activates effector pathways to enhance the immune system of mice during bacterial infection. 

### 3.6. MOTS-c Postpone Aging

The level of circulating MOTS-c decreases with age, suggesting that it may participate in regulating the process of aging-related diseases [52]. According to the studies, MOTS-c can improve the physical performance of young mice, enhance the physical capacity of old mice and improve mouse healthspan [21]. Maintaining the cell metabolic balance is regarded as the main way to prevent aging-related disease. We hypothesize that MOTS-c delay aging by regulating the expression of nuclear genes, maintaining metabolic homeostasis, and modulating cell adaptability to metabolic stress as the mechanisms mentioned above. In animal models, MOTS-c considerably delays the onset of age-related physical impairments [21]. Interestingly, it is found in studies that the longevity of the Japanese population is related to the m.1382A > C polymorphism (Table 1) [53]. Therefore, it is interesting to investigate the role of MOTS-c polymorphism of different ethnicities in longevity. This may explain the phenomenon that the Japanese have a large number of long-lived people. 

MOTS-c can raise the intracellular level of NAD^+^, whose concentration gradually declines with age [5,54]. NAD^+^ is an evolutionarily highly conserved coenzyme with multi-faceted cell functions, including energy metabolism, molecular signaling processes, epigenetic regulation, and DNA repair [55,56]. NAD^+^ levels increase under conditions that increase lifespan and healthspan, such as dietary restriction and exercise, and decrease during ageing or under conditions that decrease lifespan and healthspan, such as a high-fat diet, which supports the working model that decreased NAD^+^ levels might contribute to the ageing process [57]. On the basis of this idea, it has been predicted and validated that lower NAD^+^ levels are a shared characteristic of ageing-related disease. Therefore, MOTS-c may ameliorate the NAD^+^ shortage caused by aging-related disorders and regulate the expression of genes related to NAD^+^ concentrations [5,58]. MOTS-c can modestly increase mitochondrial respiration via the JAK pathway and enhances the expression of senescence-related secretory phenotypes in senescent cells [59]. It makes senescence cells easier to be recognized and cleared by the immune system [59,60]. It can be seen from the above discussion that an important physiological function of MOTS-c is to slow down aging, indicating that MOTS-c is a new target for detecting and treating aging-related disorders. 

## 4. Clinical Application of MOTS-c 

Currently, known physiological functions of MOTS-c include reducing insulin resistance, preventing obesity, improving muscle function, promoting bone metabolism, enhancing immune regulation, and postponing aging through the gene expression and signaling pathways described above. Table 2 summarizes the diseases associated with MOTS-c dysfunction. Table 3 summarizes the genes and pathways involved in the physiological function of MOTS-c. Based on the natural characteristics of MOTS-c, we believe that MOTS-c can be used as a diagnostic marker for metabolic diseases since the circulating MOTS-c is significantly changed in chronic fatigue syndrome, metabolic syndrome, and polycystic ovarian syndrome [61,62,63]. Studies have shown that with MOTS-c at high concentrations, the glucose uptake will be integrated into the pentose phosphate pathway to provide a substrate for the synthesis of purines [5,64]. Since glucose will not be oxidized through glycolysis, MOTS-c has fewer side effects than traditional AMPK-activating drugs and has a broader application prospect.

The development of peptide-based drugs as an effective therapeutic strategy is currently one of the hottest topics in drug development. MOTS-c is not the first active peptide discovered with a wide range of effects. Previously, the development of peptide drugs has been reported many times. The disadvantages, including low efficacy, toxicity, and drug resistance, limit the clinical application of peptide drugs [65,66,67]. Previous animal experiments have proved that MOTS-c alone can reduce obesity and insulin resistance. Therefore, we think that MOTS-c is highly effective as a peptide drug. However, the degradation of peptides through digestive enzymes, as well as the low permeability of the intestinal mucosa, are challenges for the oral delivery of peptide drugs [68,69]. It may make MOTS-c unable to be administered orally. Other problems, such as poor patient compliance with drugs and the short duration of drug effect, may occur. Therefore, enhancing the expression or function of endogenous MOTS-c may be a safer and more dependable treatment strategy. 

MOTS-c could be used to treat obesity and age-related diseases as a peptide drug. MOTS-c levels in obese people fell significantly. Interestingly, MOTS-c expression is significantly higher in brown adipose tissue (BAT) [30]. BAT is a thermogenic organ that is involved in energy expenditure and represents an attractive target to treat obesity [70]. White adipose tissue (WAT), on the other hand, functions as an endocrine organ and serves as a reservoir of energy in the form of triglycerides [71]. Obesity resulting from a chronic imbalance between energy intake and energy output with an increased WAT mass entails an increased risk of several chronic illnesses, including T2D, insulin resistance, and cardiovascular diseases (CVD) [72,73,74]. We hypothesize that MOTS-c promotes the conversion of WAT to BAT, a process known as white fat browning. As a result, raising MOTS-c levels may help to reduce obesity. MOTS-c also serves an anti-aging function. MOTS-c levels in skeletal muscle and circulation decline with age. As an exercise-induced regulator of aging metabolic homeostasis, raising the level of MOTS-c in the elderly with proper exercise can be used to prevent age-related diseases. 

The adverse effects of peptide-based drugs are a concern in clinical trials. The most common side effects of peptide-based drugs that have been reported include the production of antidrug antibodies, off-target effects, and gastrointestinal side effects. For example, anti-drug antibodies can develop in up to 40% of multiple sclerosis patients treated with IFN-β [75]. Although dipeptidyl peptidase 4 inhibitors have been approved for treating of T2D, their non-incretin substrates cause off-target effects such as heart failure [76]. The most commonly reported adverse effect of long-acting GLP-1 receptor agonists appears to be gastrointestinal effects, including nausea and vomiting [77]. In addition, patients with eosinophilic esophagitis treated with monoclonal antibodies experienced side effects such as chest pain, headache, and cough [78]. MOTS-c has several advantages over other peptide-based drugs. Firstly, since MOTS-c is an endogenous polypeptide, it is unlikely to elicit an immune response. Secondly, the off-target adverse effects will not occur at an appropriate concentration of MOTS-c. Finally, the appropriate level of MOTS-c should be non-toxic. Oral MOTS-c may cause gastrointestinal reactions, but this is a problem that all oral drugs face, so it has little bearing on MOTS-c to develop to be a highly efficient peptide drug.

## 5. Conclusions

The updated physiological functions of MOTS-c are summarized in this article. MOTS-c not only functions as a cell-autonomous peptide but also affects a number of pathological metabolic processes in the body by acting similarly to a hormone, including insulin resistance reduction, obesity prevention, muscle function improvement, bone metabolism promotion, immune regulation enhancement, and aging postponement. MOTS-c contributes to nuclear-mitochondrial signaling and has cytoprotective properties within the cells. In the cells, through inhibiting the folate cycle at the level of 5Me-THF, MOTS-c activates the AICAR-AMPK pathway. In circulation, the decreased expression of MOTS-c is observed in a number of metabolic diseases, indicating that MOTS-c is expected to become a potential molecular diagnostic marker. In this review article, we hope that by bringing attention to the potential therapeutic value of MOTS-c in the treatment and diagnosis of the metabolic disorders. 

## 6. Future Directions

Until today, there are still a lot of unanswered questions about MOTS-c waiting to be answered, despite being the current research hotspot on physiologically active peptides. Is there, for instance, any synergy or antagonism between MOTS-c and other MDPs? Will MOTS-c be used in the clinical treatment of metabolic diseases such as insulin resistance, obesity, and osteoporosis after safety testing? Is the MOTS-c polymorphism in different populations associated with obesity and longevity? What is the relationship between MOTS-c expression level and mtDNA polymorphism? To answer these questions, we need to fully understand the function of mitochondria. 

A number of studies have shown that a malfunction/degradation of mtDNA is associated with a variety of age-related diseases, including Alzheimer’s disease, macular degeneration, and cancer [79,80,81]. This malfunction/degradation of mtDNA has a progressive loss of expression of MDPs, which would reduce the regulatory peptide activities of MDPs. The tissue and circulating levels of MOTS-c fall with age; it is compelling to hypothesize that declining MDP levels are related to age-related metabolic deterioration. These mitochondrial genetic changes, we hypothesize, may contribute to the age-dependent decrease in MOTS-c levels. 

Another key question is which genes are regulated by MOTS-c entering the nucleus. Based on the characteristics of MOTS-c, as a short peptide with 16 amino acids, MOTS-c is more likely to achieve biological functions by regulating the expression of other genes. Technological advances can accelerate our understanding the function of MOTS-c. It is a good way to use the next-generation sequencing technology to find the downstream target gene of MOTS-c. The identification of MOTS-c specific binding partners and target genes may provide further insight into the coordinated network of mitochondrial-nuclear communication pathways.

## Figures and Tables

**Table 1:** The m.1382A>C polymorphism in the mtDNA region 12S rRNA (highlighted in bold) cause Lys14Gln substitution in MOTS-c.

Nucleotide Position	Nucleotide Sequence	Amino Acid (3-letter code)	Amino Acid Position	Mutation Type
1343	atg	Met	1	
1346	agg	Arg	2	
1349	tgg	Trp	3	
1352	caa	Gln	4	
1355	gaa	Glu	5	
1358	atg	Met	6	
1361	ggc	Gly	7	
1364	tac	Tyr	8	
1367	att	Ile	9	
1370	ttc	Phe	10	
1373	ta[C>T]	Tyr	11	synonymous substitution
1376	ccc	Pro	12	
1379	aga	Arg	13	
**1382**	**[A>C]aa**	**Lys>Gln**	**14**	**missense substitution**
1385	cta	Leu	15	
1388	cga	Arg	16	
1391	tag	Stop		

**Table 2:** The Diseases Associated to MOTS-c Dysfunction.

The Functions of MOTS-c	The Disease Associated to MOTS-c Dysfunction	References
Reduces insulin resistance	type 2 diabetes, diabetes-induced abnormal cardiac structures and functions	[5,23]
Prevents obesity	obesity, vascular calcification	[5,8,29]
Improves muscle function	duchenne muscular dystrophy, energy-deficient muscle illnesses	[31,32,33]
Promotes bone metabolism	osteoporosis	[45,46]
Enhances immune regulation	type 1 diabetes, multiple sclerosis, inflammation-related disorders, methicillin-resistant staphylococcus aureus infection	[48,49]
Postpones aging	aging-related diseases	[52,53,54]

**Table 3:** Genes and Pathways Involved in the Functions of MOTS-c.

The Functions of MOTS-c	The Genes and Pathways Involved in the Functions of MOTS-c	References
Reduces insulin resistance	AMPK, NRG1, ErbB4, TFAM, COX4, NRF1, GLUT4	[5,22,23,24]
Prevents obesity	AMPK, GLUT4, IL-6, TNF-α, AT-1, ET-B, PGC1α, UCP1, Dio2, ERK	[5,23,26,28,30]
MOTS-c improves muscle function	AMPK, STAT3, FOXO1, APPL1, SIRT1, PGC1α	[5,32,33,38]
Promotes bone metabolism	AMPK, TGF-β, SMAD7, OPG, RANKL, NFκB, STAT1	[45,46]
Enhances immune regulation	TCR, mTORC1, NF-κB, Nrf2, TNF-α, IL-1β, IL-6, IL-10, ERK, JNK, P38, AhR, Stat3	[26,48,50,51]
Postpones aging	JAK	[5,59]

## Abbreviations

**Abbreviation**	**Word or Phrase in Full**
5Me-THF	5-methyltetrahydro-folate
ACC	acetyl-CoA carboxylase
AMPK	AMP-activated protein kinase
AICAR	5-aminoimidazole-4-carboxamide riboside
AhR	aryl hydrocarbon receptor
AREs	antioxidant-responsive elements
APPL1	adaptor protein, phosphotyrosine interacting with PH domain and leucine zipper 1
Arg	arginine
ATP	adenosine triphosphate
AT-1	angiotensin II type 1
BAT	brown adipose tissue
COX4	cytochrome c oxidase subunit 4
CVD	cardiovascular diseases
Dio2	deiodinase 2
DMD	Duchenne muscular dystrophy
ERK	extracellular signal-regulated kinase
ErbB4	Erb-B2 receptor tyrosine kinase 4
ET-B	endothelin B
FOXO1	forkhead box protein O1
Gln	glutamine
Glu	glutamicacid
GLUT4	glucose transporter 4
GLP-1	glucagon-like peptide 1
Gly	glycine
IFN-β	interferon-β
IL-6	interleukin-6
IL-1β	interleukin-1β
IL-10	interleukin-10
Ile	isoleucine
JAK	janus activated kinase
JNK	c-Jun amino-terminal kinases
Leu	leucine
Lys	lysine
MDPs	mitochondrial-derived peptides
Met	methionine
MOTS-c	mitochondrial open reading frame of the 12S rRNA-c
mtDNA	mitochondrial DNA
mTORC1	mTOR complex 1
NAD	nicotinamide adenine dinucleotide
NRG1	neuregulin1
NRF1	nuclear respiratory factor 1
Nrf2	nuclear factor-erythroid-2-related factor 2
NF-κB	nuclear factor-kappaB
OPG	osteoprotegerin
ORF	open reading frame
PGC1α	peroxisome proliferator-activated receptor-γ coactivator 1α
Phe	phenylalanine
Pro	proline
RANKL	receptor activator of nuclear factor kappa-B ligand
ROS	reactive oxygen species
rRNA	ribosomal RNA
SHLP	small humanin-like peptides
Smad	sekelsky mothers against decapentaplegic
SIRT1	sirtuin 1
STAT1	signal transducer and activator of transcription 1
STAT3	signal transducer and activator of transcription 3
T1D	type 1 diabetes
T2D	type 2 diabetes
TCR	T cell receptor
TFAM	transcription factor A for mitochondria
TNF-α	tumor necrosis factor-α
TGF-β	transforming growth factor-β
tRNA	transfer RNA
Trp	tryptophan
Tyr	tyrosine
UCP1	uncoupling protein-1
WAT	white adipose tissue
